# 4-[2-(4-Meth­oxy­phen­yl)eth­yl]-3-(thio­phen-2-ylmeth­yl)-1*H*-1,2,4-triazol-5(4*H*)-one monohydrate

**DOI:** 10.1107/S1600536811045508

**Published:** 2011-11-05

**Authors:** Anuradha Gurumoorthy, Vasuki Gopalsamy, K. Ramamurthi, Dilek Ünlüer, Fatih Çelik

**Affiliations:** a Department of Physics, Saveetha School of Engineering, Saveetha University, Chennai 600 077, India; b Department of Physics, Kunthavai Naachiar Government Arts College (w) (Autonomous), Thanjavur 613 007, India; c Crystal Growth and Thin Film Laboratory, School of Physics, Bharathidasan University, Tiruchirappalli 620 024, India; d Department of Chemistry, Faculty of Arts and Sciences, Karadeniz Teknik University, Trabzon 61080, Turkey

## Abstract

In the title compound, C_16_H_17_N_3_O_2_S·H_2_O, the triazole ring makes a dihedral angle of 34.63 (6)° with the benzene ring. The thio­phene ring is disordered over two orientations [occupancy ratio = 0.634 (4):0.366 (4)] which make dihedral angles of 54.61 (16) and 54.57 (31)° with the triazole ring. Inter­molecular N—H⋯O and O—H⋯O hydrogen bonds stabilize the crystal structure.

## Related literature

For the biological activity of triazoles, see: Ünver *et al.* (2006 ▶); Ustabaş *et al.* (2007 ▶). For related structures, see: Ünver *et al.* (2006 ▶, 2010 ▶); Yılmaz *et al.* (2006 ▶). For the synthesis, see: Ünver *et al.* (2011 ▶).
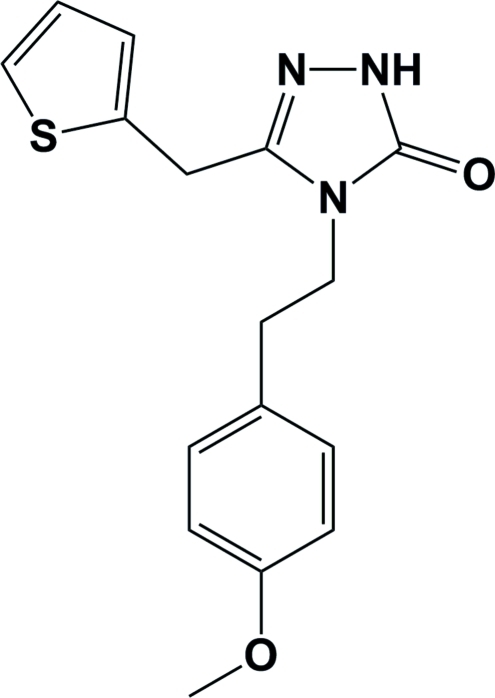


## Experimental

### 

#### Crystal data

C_16_H_17_N_3_O_2_S·H_2_O
*M* *_r_* = 333.40
Monoclinic, 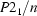

*a* = 6.7945 (1) Å
*b* = 30.8791 (7) Å
*c* = 7.8564 (2) Å
β = 102.057 (1)°
*V* = 1611.97 (6) Å^3^
*Z* = 4
Mo *K*α radiation
μ = 0.22 mm^−1^
*T* = 292 K
0.30 × 0.25 × 0.20 mm

#### Data collection

Bruker APEXII KappaCCD diffractometer
Absorption correction: multi-scan (*SADABS*; Bruker, 2004 ▶) *T*_min_ = 0.901, *T*_max_ = 0.962
30445 measured reflections
3176 independent reflections
2877 reflections with *I* > 2σ(*I*)
*R*_int_ = 0.024

#### Refinement

*R*[*F* ^2^ > 2σ(*F* ^2^)] = 0.036
*wR*(*F* ^2^) = 0.090
*S* = 1.08
3176 reflections
240 parameters
H atoms treated by a mixture of independent and constrained refinement
Δρ_max_ = 0.16 e Å^−3^
Δρ_min_ = −0.23 e Å^−3^

### 

Data collection: *APEX2* (Bruker, 2004 ▶); cell refinement: *SAINT-Plus* (Bruker, 2004 ▶); data reduction: *SAINT-Plus*; program(s) used to solve structure: *SIR92* (Altomare *et al.*, 1993 ▶); program(s) used to refine structure: *SHELXL97* (Sheldrick, 2008 ▶); molecular graphics: *ORTEP-3* (Farrugia, 1997 ▶) and *PLATON* (Spek, 2009 ▶); software used to prepare material for publication: *SHELXL97*.

## Supplementary Material

Crystal structure: contains datablock(s) I, global. DOI: 10.1107/S1600536811045508/su2321sup1.cif

Structure factors: contains datablock(s) I. DOI: 10.1107/S1600536811045508/su2321Isup2.hkl

Supplementary material file. DOI: 10.1107/S1600536811045508/su2321Isup3.cml

Additional supplementary materials:  crystallographic information; 3D view; checkCIF report

## Figures and Tables

**Table 1:** Hydrogen-bond geometry (Å, °)

*D*—H⋯*A*	*D*—H	H⋯*A*	*D*⋯*A*	*D*—H⋯*A*
N2—H2*A*⋯O3^i^	0.867 (19)	1.975 (19)	2.8334 (16)	170.3 (16)
O3—H3*A*⋯O1^ii^	0.85 (2)	1.95 (2)	2.7679 (15)	163.6 (19)
O3—H3*B*⋯O1^iii^	0.86 (2)	1.97 (2)	2.8231 (15)	175 (2)

Symmetry codes: (i) 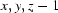; (ii) 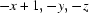; (iii) 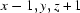.

**Fig. 1.**
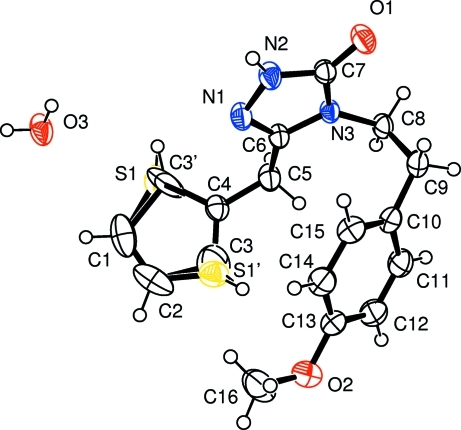
The molecular structure of the title compound, showing the crystallographic numbering scheme and displacement ellipsoids drawn at the 50% probability level.

**Fig. 2.**
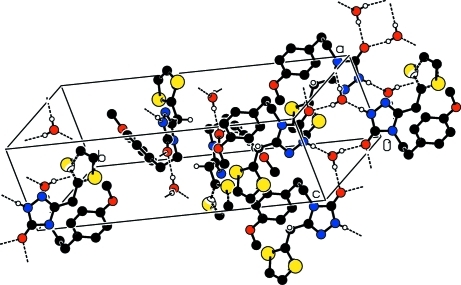
A partial view of the crystal packing of the title compound with the intermolecular N—H···O and O—H···O hydrogen bonds shown as dashed lines (see Table 1 for details).

## supplementary crystallographic information

### Comment

1,2,4-triazole derivatives have a broad-spectrum of biological effects, such as insecticidal, herbicidal, antitumor and plant growth regulatory activities. Di- or tri-substituted 1,2,4-triazole derivatives have also been reported to show antituberculotic and antimicrobial activities (Ustabaş *et al.*, 2007). Various 1,2,4-triazole derivatives have been reported as showing fungicidal and antimicrobial activitry as well as having applications as anticonvulsants and antidepressants (Ünver *et al.*, 2006). In view of the importance of triazole compounds, and in order to examine the structure activity of 1,2,4-triazole with a thiophene and a methylphenyl substituent, we prepared the title compound and report herein on its crystal structure.

The title compound crystallizes as a monohydrate, Fig. 1. The bond lengths and angles are within normal ranges (Yılmaz *et al.*, 2006; Ünver *et al.*, 2010). It contains three planar rings namely, a triazole ring [A = (N1,N2,C7,N3,C6)], a benzene ring [B = (C10—C15)] and a thiophene ring [C = (C1—C4,S1]. The dihedral angles between the mean planes of the rings A/B, A/C and B/C are 34.63 (6)°, 54.61 (16)° and 54.64 (13)°, respectively. Atom N3 has a trigonal configuration, the sum of three bond angles around it being 360 °.

The thiophene ring is disordered over two orientations [occupancy ratio = 0.634 (4)/0.366 (4)] with respect to the C4—C5 bond; the dihedral angles between the triazole and the two orientations of the and the thiophene ring are 54.61 (16)Å and 54.57 (31) Å, respectively. The N3—C8—C9—C10 torsion angle of 64.61 (14)° indicates that the triazole ring and the benzene ring are substituted equatorially across bond C8—C9. The widening of exocyclic angle C5—C4—C3 [124.1 (6)°] from the normal value of 120°, may be due to the steric repulsion between atoms H3A and H5A (H3A···H5A = 2.5354 (0) Å).

In the crystal, the water molecule is involved in intermolecular O—H···N and O—H···O hydrogen bonding (Fig. 2 and Table 1), which are effective in stabilizing the crystal structure.

### Experimental

The compound was synthesized according to the published procedure (Ünver *et al.*, 2011).

### Refinement

NH atom and water H atoms were located in a difference Fourier map and were freely refined. The C-bound H atoms were positioned geometrically and treated as riding atoms: C—H = 0.93, 0.96 and 0.97 Å for CH, CH_3_ and CH_2_ H atoms, respectively, with *U*_iso_(H) = k × *U*_eq_(C), where k = 1.5 for CH_3_ H atoms, and k = 2 for all other H atoms.

### Crystal data

**Table:**

C_16_H_17_N_3_O_2_S·H_2_O	*F*(000) = 704
*M**_r_* = 333.40	*D*_x_ = 1.374 Mg m^−^^3^
Monoclinic, *P*2_1_/*n*	Mo *K*α radiation, λ = 0.71073 Å
Hall symbol: -P 2yn	Cell parameters from 7004 reflections
*a* = 6.7945 (1) Å	θ = 2.6–32.1°
*b* = 30.8791 (7) Å	µ = 0.22 mm^−^^1^
*c* = 7.8564 (2) Å	*T* = 292 K
β = 102.057 (1)°	Block, colourless
*V* = 1611.97 (6) Å^3^	0.30 × 0.25 × 0.20 mm
*Z* = 4	

### Data collection

**Table:**

Bruker APEXII KappaCCD diffractometer	3176 independent reflections
Radiation source: fine-focus sealed tube	2877 reflections with *I* > 2σ(*I*)
graphite	*R*_int_ = 0.024
ω and φ scan	θ_max_ = 26.0°, θ_min_ = 2.6°
Absorption correction: multi-scan (*SADABS*; Bruker, 2004)	*h* = −8→8
*T*_min_ = 0.901, *T*_max_ = 0.962	*k* = −38→38
30445 measured reflections	*l* = −9→9

### Refinement

**Table:**

Refinement on *F*^2^	Primary atom site location: structure-invariant direct methods
Least-squares matrix: full	Secondary atom site location: difference Fourier map
*R*[*F*^2^ > 2σ(*F*^2^)] = 0.036	Hydrogen site location: inferred from neighbouring sites
*wR*(*F*^2^) = 0.090	H atoms treated by a mixture of independent and constrained refinement
*S* = 1.08	*w* = 1/[σ^2^(*F*_o_^2^) + (0.0386*P*)^2^ + 0.4907*P*] where *P* = (*F*_o_^2^ + 2*F*_c_^2^)/3
3176 reflections	(Δ/σ)_max_ < 0.001
240 parameters	Δρ_max_ = 0.16 e Å^−^^3^
0 restraints	Δρ_min_ = −0.23 e Å^−^^3^

### Special details

**Table:**

Geometry. All e.s.d.'s (except the e.s.d. in the dihedral angle between two l.s. planes) are estimated using the full covariance matrix. The cell e.s.d.'s are taken into account individually in the estimation of e.s.d.'s in distances, angles and torsion angles; correlations between e.s.d.'s in cell parameters are only used when they are defined by crystal symmetry. An approximate (isotropic) treatment of cell e.s.d.'s is used for estimating e.s.d.'s involving l.s. planes.
Refinement. Refinement of *F*^2^ against ALL reflections. The weighted *R*-factor *wR* and goodness of fit *S* are based on *F*^2^, conventional *R*-factors *R* are based on *F*, with *F* set to zero for negative *F*^2^. The threshold expression of *F*^2^ > σ(*F*^2^) is used only for calculating *R*-factors(gt) *etc*. and is not relevant to the choice of reflections for refinement. *R*-factors based on *F*^2^ are statistically about twice as large as those based on *F*, and *R*- factors based on ALL data will be even larger.

### Fractional atomic coordinates and isotropic or equivalent isotropic displacement parameters (Å^2^)

**Table:**

	*x*	*y*	*z*	*U*_iso_*/*U*_eq_	Occ. (<1)
S1	0.5309 (3)	0.08013 (7)	0.3984 (2)	0.0486 (3)	0.634 (4)
C3	0.7012 (18)	0.1520 (3)	0.3706 (15)	0.083 (5)	0.634 (4)
H3	0.7865	0.1737	0.3470	0.099*	0.634 (4)
S1'	0.7024 (6)	0.15919 (17)	0.3771 (5)	0.0490 (6)	0.366 (4)
C3'	0.562 (2)	0.0828 (5)	0.386 (2)	0.083 (7)	0.366 (4)
H3'	0.5387	0.0531	0.3765	0.100*	0.366 (4)
O1	0.86927 (14)	0.01851 (4)	−0.29652 (13)	0.0451 (3)	
O2	0.83597 (18)	0.25350 (4)	0.13401 (18)	0.0602 (3)	
N1	0.61110 (16)	0.05605 (4)	0.01137 (15)	0.0377 (3)	
N2	0.62048 (17)	0.03584 (4)	−0.14363 (15)	0.0381 (3)	
H2A	0.510 (3)	0.0281 (6)	−0.213 (2)	0.052 (5)*	
N3	0.92215 (15)	0.05475 (3)	−0.03060 (13)	0.0293 (2)	
C1	0.4375 (3)	0.12019 (8)	0.4753 (2)	0.0672 (6)	
H1	0.3274	0.1176	0.5278	0.081*	
C2	0.5251 (3)	0.15777 (7)	0.4608 (2)	0.0710 (6)	
H2	0.4850	0.1841	0.5001	0.085*	
C4	0.7112 (2)	0.10794 (5)	0.32991 (17)	0.0362 (3)	
C5	0.8700 (2)	0.08860 (5)	0.24723 (18)	0.0390 (3)	
H5A	0.9634	0.1113	0.2317	0.047*	
H5B	0.9446	0.0675	0.3267	0.047*	
C6	0.79510 (18)	0.06713 (4)	0.07565 (16)	0.0311 (3)	
C7	0.80746 (18)	0.03455 (4)	−0.17298 (16)	0.0319 (3)	
C8	1.13309 (17)	0.06594 (4)	−0.02062 (17)	0.0328 (3)	
H8A	1.2025	0.0414	−0.0577	0.039*	
H8B	1.1963	0.0725	0.0991	0.039*	
C9	1.1530 (2)	0.10497 (5)	−0.13524 (19)	0.0384 (3)	
H9A	1.2942	0.1096	−0.1351	0.046*	
H9B	1.0846	0.0987	−0.2539	0.046*	
C10	1.0665 (2)	0.14568 (4)	−0.07543 (17)	0.0352 (3)	
C11	1.1851 (2)	0.17298 (5)	0.0446 (2)	0.0442 (3)	
H11	1.3213	0.1669	0.0821	0.053*	
C12	1.1050 (2)	0.20875 (5)	0.1088 (2)	0.0493 (4)	
H12	1.1876	0.2268	0.1878	0.059*	
C13	0.9017 (2)	0.21811 (4)	0.0567 (2)	0.0422 (3)	
C14	0.7812 (2)	0.19195 (5)	−0.0651 (2)	0.0430 (3)	
H14	0.6453	0.1983	−0.1032	0.052*	
C15	0.8653 (2)	0.15618 (5)	−0.12984 (18)	0.0397 (3)	
H15	0.7839	0.1388	−0.2123	0.048*	
C16	0.6266 (3)	0.26164 (7)	0.1030 (3)	0.0679 (5)	
H16A	0.6017	0.2865	0.1685	0.102*	
H16B	0.5590	0.2369	0.1382	0.102*	
H16C	0.5769	0.2669	−0.0188	0.102*	
O3	0.25229 (16)	0.02104 (4)	0.61748 (16)	0.0549 (3)	
H3A	0.240 (3)	0.0083 (7)	0.520 (3)	0.065 (6)*	
H3B	0.139 (3)	0.0193 (7)	0.649 (3)	0.072 (6)*	

### Atomic displacement parameters (Å^2^)

**Table:**

	*U*^11^	*U*^22^	*U*^33^	*U*^12^	*U*^13^	*U*^23^
S1	0.0394 (4)	0.0651 (8)	0.0458 (5)	−0.0037 (4)	0.0195 (4)	0.0000 (5)
C3	0.091 (6)	0.096 (10)	0.068 (5)	0.002 (4)	0.033 (4)	0.006 (4)
S1'	0.0614 (13)	0.0430 (8)	0.0430 (12)	−0.0015 (7)	0.0118 (9)	−0.0045 (7)
C3'	0.085 (10)	0.076 (9)	0.094 (9)	0.029 (7)	0.028 (5)	−0.040 (7)
O1	0.0340 (5)	0.0615 (7)	0.0416 (6)	−0.0025 (4)	0.0119 (4)	−0.0186 (5)
O2	0.0593 (7)	0.0431 (6)	0.0833 (9)	−0.0012 (5)	0.0266 (6)	−0.0127 (6)
N1	0.0279 (5)	0.0508 (7)	0.0362 (6)	−0.0035 (5)	0.0108 (5)	−0.0084 (5)
N2	0.0257 (5)	0.0530 (7)	0.0357 (6)	−0.0059 (5)	0.0066 (5)	−0.0119 (5)
N3	0.0225 (5)	0.0348 (5)	0.0313 (5)	−0.0004 (4)	0.0075 (4)	−0.0024 (4)
C1	0.0442 (9)	0.1205 (19)	0.0389 (9)	0.0111 (10)	0.0136 (7)	0.0021 (10)
C2	0.0837 (14)	0.0734 (13)	0.0498 (10)	0.0374 (11)	0.0000 (10)	−0.0188 (9)
C4	0.0367 (7)	0.0444 (8)	0.0279 (6)	−0.0004 (6)	0.0079 (5)	−0.0030 (6)
C5	0.0308 (6)	0.0527 (8)	0.0343 (7)	−0.0037 (6)	0.0087 (5)	−0.0071 (6)
C6	0.0260 (6)	0.0363 (7)	0.0324 (6)	0.0003 (5)	0.0093 (5)	−0.0006 (5)
C7	0.0274 (6)	0.0362 (7)	0.0328 (6)	−0.0010 (5)	0.0078 (5)	−0.0039 (5)
C8	0.0211 (6)	0.0394 (7)	0.0383 (7)	0.0006 (5)	0.0072 (5)	−0.0027 (5)
C9	0.0320 (6)	0.0437 (8)	0.0426 (7)	−0.0035 (6)	0.0149 (6)	−0.0003 (6)
C10	0.0348 (6)	0.0358 (7)	0.0363 (7)	−0.0040 (5)	0.0106 (5)	0.0049 (5)
C11	0.0315 (7)	0.0500 (8)	0.0504 (8)	−0.0054 (6)	0.0072 (6)	−0.0027 (7)
C12	0.0440 (8)	0.0482 (9)	0.0559 (9)	−0.0123 (7)	0.0105 (7)	−0.0120 (7)
C13	0.0481 (8)	0.0329 (7)	0.0496 (8)	−0.0030 (6)	0.0191 (7)	0.0030 (6)
C14	0.0354 (7)	0.0418 (8)	0.0500 (8)	0.0038 (6)	0.0047 (6)	0.0083 (6)
C15	0.0377 (7)	0.0396 (7)	0.0390 (7)	−0.0022 (6)	0.0013 (6)	0.0024 (6)
C16	0.0661 (11)	0.0635 (11)	0.0783 (13)	0.0186 (9)	0.0249 (10)	−0.0066 (10)
O3	0.0328 (5)	0.0859 (9)	0.0473 (6)	−0.0090 (5)	0.0111 (5)	−0.0282 (6)

### Geometric parameters (Å, °)

**Table:**

S1—C1	1.568 (3)	C5—C6	1.4930 (18)
S1—C4	1.674 (2)	C5—H5A	0.9700
C3—C4	1.402 (10)	C5—H5B	0.9700
C3—C2	1.523 (13)	C8—C9	1.5273 (19)
C3—H3	0.9300	C8—H8A	0.9700
S1'—C2	1.488 (6)	C8—H8B	0.9700
S1'—C4	1.630 (5)	C9—C10	1.5037 (19)
C3'—C4	1.422 (14)	C9—H9A	0.9700
C3'—C1	1.666 (14)	C9—H9B	0.9700
C3'—H3'	0.9300	C10—C15	1.3833 (19)
O1—C7	1.2386 (15)	C10—C11	1.390 (2)
O2—C13	1.3695 (18)	C11—C12	1.373 (2)
O2—C16	1.415 (2)	C11—H11	0.9300
N1—C6	1.2928 (16)	C12—C13	1.387 (2)
N1—N2	1.3817 (16)	C12—H12	0.9300
N2—C7	1.3383 (16)	C13—C14	1.382 (2)
N2—H2A	0.867 (19)	C14—C15	1.388 (2)
N3—C7	1.3724 (16)	C14—H14	0.9300
N3—C6	1.3745 (16)	C15—H15	0.9300
N3—C8	1.4604 (15)	C16—H16A	0.9600
C1—C2	1.320 (3)	C16—H16B	0.9600
C1—H1	0.9300	C16—H16C	0.9600
C2—H2	0.9300	O3—H3A	0.85 (2)
C4—C5	1.4955 (18)	O3—H3B	0.86 (2)
			
C1—S1—C4	95.81 (15)	N1—C6—N3	111.59 (11)
C4—C3—C2	107.5 (8)	N1—C6—C5	126.14 (11)
C4—C3—H3	126.3	N3—C6—C5	122.20 (11)
C2—C3—H3	126.3	O1—C7—N2	129.52 (12)
C2—S1'—C4	98.2 (3)	O1—C7—N3	126.36 (11)
C4—C3'—C1	102.1 (9)	N2—C7—N3	104.12 (11)
C4—C3'—H3'	128.9	N3—C8—C9	111.22 (10)
C1—C3'—H3'	128.9	N3—C8—H8A	109.4
C13—O2—C16	118.32 (14)	C9—C8—H8A	109.4
C6—N1—N2	104.18 (10)	N3—C8—H8B	109.4
C7—N2—N1	112.67 (11)	C9—C8—H8B	109.4
C7—N2—H2A	127.8 (11)	H8A—C8—H8B	108.0
N1—N2—H2A	119.2 (11)	C10—C9—C8	112.76 (11)
C7—N3—C6	107.44 (10)	C10—C9—H9A	109.0
C7—N3—C8	122.35 (10)	C8—C9—H9A	109.0
C6—N3—C8	129.45 (11)	C10—C9—H9B	109.0
C2—C1—S1	115.75 (16)	C8—C9—H9B	109.0
C2—C1—C3'	107.2 (5)	H9A—C9—H9B	107.8
S1—C1—C3'	8.5 (5)	C15—C10—C11	117.57 (13)
C2—C1—H1	122.1	C15—C10—C9	121.67 (12)
S1—C1—H1	122.1	C11—C10—C9	120.67 (12)
C3'—C1—H1	130.6	C12—C11—C10	121.30 (13)
C1—C2—S1'	119.0 (2)	C12—C11—H11	119.4
C1—C2—C3	110.4 (4)	C10—C11—H11	119.4
S1'—C2—C3	8.6 (6)	C11—C12—C13	120.37 (14)
C1—C2—H2	124.8	C11—C12—H12	119.8
S1'—C2—H2	116.2	C13—C12—H12	119.8
C3—C2—H2	124.8	O2—C13—C14	124.94 (14)
C3—C4—C3'	112.7 (8)	O2—C13—C12	115.59 (14)
C3—C4—C5	124.1 (6)	C14—C13—C12	119.47 (14)
C3'—C4—C5	123.1 (6)	C13—C14—C15	119.30 (13)
C3—C4—S1'	0.7 (7)	C13—C14—H14	120.3
C3'—C4—S1'	113.3 (6)	C15—C14—H14	120.3
C5—C4—S1'	123.4 (2)	C10—C15—C14	121.95 (13)
C3—C4—S1	110.5 (6)	C10—C15—H15	119.0
C3'—C4—S1	2.2 (7)	C14—C15—H15	119.0
C5—C4—S1	125.30 (14)	O2—C16—H16A	109.5
S1'—C4—S1	111.2 (2)	O2—C16—H16B	109.5
C6—C5—C4	115.37 (11)	H16A—C16—H16B	109.5
C6—C5—H5A	108.4	O2—C16—H16C	109.5
C4—C5—H5A	108.4	H16A—C16—H16C	109.5
C6—C5—H5B	108.4	H16B—C16—H16C	109.5
C4—C5—H5B	108.4	H3A—O3—H3B	107.9 (19)
H5A—C5—H5B	107.5		
			
C6—N1—N2—C7	0.15 (16)	S1—C4—C5—C6	64.33 (18)
C4—S1—C1—C2	−0.15 (19)	N2—N1—C6—N3	−0.32 (15)
C4—S1—C1—C3'	−2(4)	N2—N1—C6—C5	−177.25 (13)
C4—C3'—C1—C2	−0.6 (9)	C7—N3—C6—N1	0.38 (15)
C4—C3'—C1—S1	178 (5)	C8—N3—C6—N1	170.39 (12)
S1—C1—C2—S1'	−0.8 (3)	C7—N3—C6—C5	177.45 (12)
C3'—C1—C2—S1'	−0.5 (6)	C8—N3—C6—C5	−12.5 (2)
S1—C1—C2—C3	−0.3 (5)	C4—C5—C6—N1	−15.2 (2)
C3'—C1—C2—C3	−0.1 (7)	C4—C5—C6—N3	168.18 (12)
C4—S1'—C2—C1	1.2 (3)	N1—N2—C7—O1	178.89 (14)
C4—S1'—C2—C3	−1(4)	N1—N2—C7—N3	0.07 (15)
C4—C3—C2—C1	0.7 (8)	C6—N3—C7—O1	−179.12 (13)
C4—C3—C2—S1'	178 (4)	C8—N3—C7—O1	10.0 (2)
C2—C3—C4—C3'	−1.2 (10)	C6—N3—C7—N2	−0.25 (14)
C2—C3—C4—C5	−177.1 (3)	C8—N3—C7—N2	−171.14 (11)
C2—C3—C4—S1'	−157.00 (5)	C7—N3—C8—C9	74.50 (15)
C2—C3—C4—S1	−0.8 (7)	C6—N3—C8—C9	−94.21 (15)
C1—C3'—C4—C3	1.1 (11)	N3—C8—C9—C10	64.61 (14)
C1—C3'—C4—C5	177.1 (3)	C8—C9—C10—C15	−87.12 (16)
C1—C3'—C4—S1'	1.4 (10)	C8—C9—C10—C11	89.39 (15)
C1—C3'—C4—S1	−8(17)	C15—C10—C11—C12	0.9 (2)
C2—S1'—C4—C3	23.00 (3)	C9—C10—C11—C12	−175.74 (14)
C2—S1'—C4—C3'	−1.6 (7)	C10—C11—C12—C13	0.9 (2)
C2—S1'—C4—C5	−177.30 (15)	C16—O2—C13—C14	7.3 (2)
C2—S1'—C4—S1	−1.2 (2)	C16—O2—C13—C12	−172.27 (16)
C1—S1—C4—C3	0.6 (5)	C11—C12—C13—O2	177.43 (14)
C1—S1—C4—C3'	172 (18)	C11—C12—C13—C14	−2.2 (2)
C1—S1—C4—C5	176.87 (13)	O2—C13—C14—C15	−178.05 (14)
C1—S1—C4—S1'	0.9 (2)	C12—C13—C14—C15	1.5 (2)
C3—C4—C5—C6	−119.9 (6)	C11—C10—C15—C14	−1.6 (2)
C3'—C4—C5—C6	64.6 (8)	C9—C10—C15—C14	175.04 (13)
S1'—C4—C5—C6	−120.2 (2)	C13—C14—C15—C10	0.4 (2)

### Hydrogen-bond geometry (Å, °)

**Table:**

*D*—H···*A*	*D*—H	H···*A*	*D*···*A*	*D*—H···*A*
N2—H2A···O3^i^	0.867 (19)	1.975 (19)	2.8334 (16)	170.3 (16)
O3—H3A···O1^ii^	0.85 (2)	1.95 (2)	2.7679 (15)	163.6 (19)
O3—H3B···O1^iii^	0.86 (2)	1.97 (2)	2.8231 (15)	175 (2)

Symmetry codes: (i) *x*, *y*, *z*−1; (ii) −*x*+1, −*y*, −*z*; (iii) *x*−1, *y*, *z*+1.
